# Rudolf Zaugger

**Published:** 1884-07

**Authors:** 


RUDOLF ZAUGGER, Director of the Zurich Veterinary School, was born in the canton of Zurich on the 30th of November, 1836. He was the son of a well-to-do agriculturist. He studied at Zurich Veterinary School under Wirth and Hirzel, and was much admired for his diligence. He graduated in 1845 with honor. He went into practice, to soon return to Zurich, where he attended lectures at the University. Having received assistance from the State for the purpose, he went to study at the schools at Lyons and Toulouse. On Hirzel's death he was appointed Chief Teacher and Director of the Zurich School, which positions he filled with great honor to himself and benefit to his country, which conferred many honors upon him. He was very influential in advancing the position of the veterinarian in the army as well as in the various civil positions in the country, and in devising the organization of a veterinary police system. He was President of the Swiss Veterinary Society for a long time, as well as Editor of the "Archiv für Thierheilkunde," and also was elected President of the Veterinary Congress at Zurich in 1867. He was quite an important politician, being known as a leader among the Swiss Democrats, and was active in all the duties of a public spirited citizen.—*Deutsche Zeitschrift für Thiermedicin*, 1883, *Part I.*

